# Influence of an Exercise-Specific Face Mask on Physiological and Perceptual Responses to Graded Exercise

**DOI:** 10.3390/jfmk9010048

**Published:** 2024-03-08

**Authors:** Aidan K. Comeau, Kelvin E. Jones, Eric C. Parent, Michael D. Kennedy

**Affiliations:** 1 Faculty of Kinesiology, Sport, and Recreation, College of Health Sciences, University of Alberta, Edmonton, AB T6G 2R3, Canadakejones@ualberta.ca (K.E.J.); 2 Department of Physical Therapy, Faculty of Rehabilitation Medicine, College of Health Sciences, University of Alberta, Edmonton, AB T6G 2R3, Canada; eparent@ualberta.ca

**Keywords:** aerobic exercise, dyspnea, respiratory function, exercise-induced bronchoconstriction, exercise performance, perceived exertion

## Abstract

The impact of exercise-specific face masks (ESFMs) in aerobically fit individuals on physiological, perceptual, respiratory, and performance responses remains unclear. How ESFMs mitigate exercise-induced bronchoconstriction (EIB) is also unknown. Thus, this study aimed to determine how an ESFM altered within-exercise physiological, perceptual, respiratory, and performance responses to graded treadmill exercise. Twenty-four individuals (11 females) completed a discontinuous graded exercise test on a treadmill under two conditions (ESFM and unmasked). Physiological, respiratory function, and perceptual measures were assessed. Performance was determined by time to exhaustion. Statistical analyses included linear mixed-effects modeling, repeated measures analysis of variance, and pairwise comparisons using an alpha value of 0.05. ESFM use significantly impaired performance (median = −150.5 s) and decreased arterial oxygen saturation at maximal intensity (mean = −3.7%). Perceptions of air hunger and work of breathing were elevated across submaximal and maximal intensities. Perceived exertion and breathing discomfort were significantly elevated submaximally but not maximally. Spirometry measures were not significantly different at termination but were significantly improved at submaximal intensities in participants with and without EIB. ESFM use in fit individuals increased perceptual discomfort, impaired performance, and augmented arterial desaturation. Respiratory function improvements were observed but were accompanied by adverse perceptual sensations. Despite this, performance impairments may limit the real-world utility of ESFMs for athletes.

## 1. Introduction

As the use of a protective face mask (PFM) was recommended in public settings to mitigate the spread of Severe Acute Respiratory Syndrome Coronavirus II during the COVID-19 pandemic, research emerged attempting to elucidate the potential effects of PFM use during exercise. Collectively, understanding of a PFM’s influence on physiological and perceptual responses during exercise is much improved, where although a PFM may acutely modify cardiorespiratory and perceptual responses, they pose little risk to health during exercise [1]. However, the extent to which physiological parameters are modified during exercise remains disputed, with conflicting findings in recent studies [2]. The heterogeneity in research findings is likely attributable to several factors, including differences in mask type, participant characteristics, exercise modality and intensity, as well as environmental characteristics [1]. Studies that have evaluated metabolic responses via indirect calorimetry, although insightful, also likely impair our ability to understand the real-world responses to PFM use during exercise [3,4,5]. This is due to a “double masked” arrangement (metabolic collection mask secured over top of the PFM being assessed), which may confound measurement of breathing dynamics and perceptual discomfort beyond the contribution of the PFM alone.

All PFMs impose a combination of added breathing resistance and dead space, with the degree to which they do this varying between different types of PFMs [6]. These factors likely impact ventilatory and cardiopulmonary responses to exercise by increasing expired air rebreathing and elevating breathing resistance [1,6]. Additionally, these factors may influence one’s rating of perceived exertion (RPE) and/or dyspnea during exercise, which may, in turn, have a detrimental impact on exercise tolerance and performance [7]. Although the influence may be negligible at rest or during light-intensity physical activity [8], the elevated metabolic demands associated with vigorous-intensity physical activity may accentuate the physiological and perceptual burden. For aerobically fit individuals, whose high level of cardiovascular fitness coincides with heightened ventilatory demands, this influence is likely magnified. Given that work of breathing (WOB) increases exponentially with increased exercise intensity and associated ventilation [9], athletes and other aerobically fit individuals may experience elevated physiological and perceptual responses during exercise, especially during high-intensity activity.

Exercise-specific face masks (ESFMs) are a novel type of PFM that boast low resistance to airflow and suitability for exercise across a range of intensities [10]. Given that the properties of these PFMs differ significantly from clinical PFMs such as surgical masks and N95 respirators, their influence on physiological and perceptual responses may differ as well. Although claims surrounding their acceptability across a spectrum of exercise intensities have been made, few studies have been conducted assessing PFMs designed specifically for exercise [3,11]. Thus, it remains unclear if ESFMs impose a substantial physiological and/or perceptual burden, particularly in aerobically fit individuals exercising at submaximal and maximal intensities.

Face coverings have also been used to ameliorate the negative effects of hyperpnea in cool and cold air conditions due to the known benefits of heating and humidification of inspired air, which can attenuate cold air exercise-induced bronchoconstriction (EIB) [12,13]. Specifically, inspired air in cold air conditions has near-zero water content, and this dry air is a primary stimulus for EIB [14]. Thus, an ESFM may help to protect against airway desiccation when exercising in dry environments, leading to less airway narrowing post-exercise, especially in individuals with airway hyperresponsiveness [15]. Types of mouth coverings that have attenuated post-exercise bronchoconstriction in cold air include a scarf worn over the mouth [16] or a simple surgical mask worn in asthmatic children [15]. How an ESFM might reduce EIB in aerobically fit individuals with no underlying asthmatic conditions is unknown. There is also limited evidence on when within-session changes in respiratory function might occur, often referred to as breakthrough EIB [17,18], and whether breakthrough EIB affects the magnitude of post-exercise reductions in respiratory function. Currently, no research has investigated whether an ESFM affects breakthrough EIB during aerobic exercise of varying intensities.

Thus, this study aimed to determine if the use of an ESFM in aerobically fit individuals accustomed to intense exercise significantly altered within-exercise physiological, perceptual, respiratory, and performance responses to graded treadmill exercise. It was hypothesized that the use of an ESFM would increase the within-exercise perceptual burden without influencing physiological responses to a given exercise intensity, impairing peak exercise performance. Additionally, we hypothesized that the use of an ESFM would help to maintain within-exercise respiratory function in individuals prone to changes in airway caliber, especially those with EIB.

## 2. Materials and Methods

### 2.1. Participants

Twenty-four aerobically fit individuals (11 females) were recruited for this study. This sample size exceeded that required for 80% power, with an alpha = 0.05 and a standard mean difference of 0.60, based on a T-distribution (n = 19) (GPower 3.1.9.7, Kiel, Germany). This effect size was estimated based on a meta-analysis by Shaw et al. [19], who found standard mean differences for dyspnea of +0.60 when a PFM was worn during exercise. Other outcome measures were carefully considered; however, we decided on dyspnea because perceptions and sensations play a crucial role in understanding the use of PFMs during exercise. Dyspnea is a key measure related to perceptions and sensations associated with PFM use and has been evaluated before in other peer-reviewed research. Thus, we used dyspnea as our measure to determine that a sample size of >20 provided sufficient power to observe any differences in the primary outcome measure of dyspnea.

A convenience sample of healthy, aerobically fit individuals between the ages of 18 and 40 with a minimum of 3 years of regular aerobic exercise training was recruited. Participants who experienced exercise-related reductions in respiratory function but were otherwise healthy were included, as EIB is known to affect a high proportion of athletes [20]. Participants were required to complete the Canadian Society for Exercise Physiology “Get Active” Questionnaire as the primary screen to ensure they could safely undertake exercise [21]. All subjects gave their informed consent for inclusion before they participated in the study. The study was conducted in accordance with the Declaration of Helsinki, and the protocol was approved by the University of Alberta Research Ethics Board (Pro00107700).

### 2.2. Design Overview

**Figure 1:**
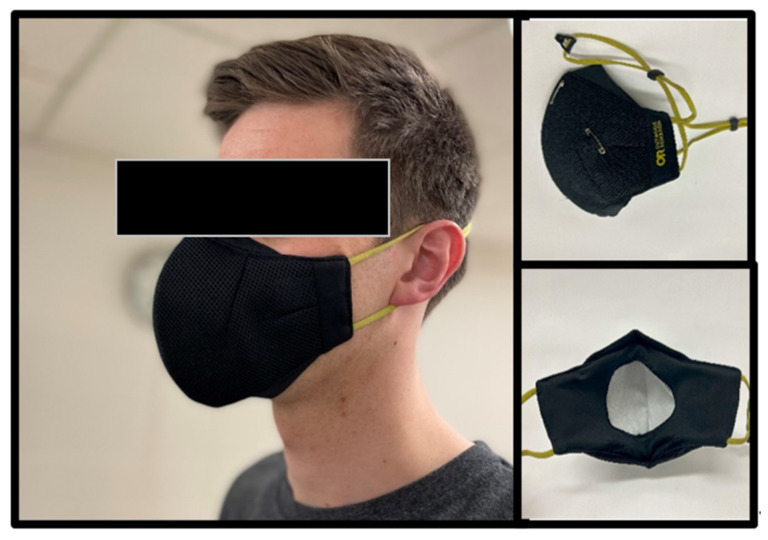
Outdoor Research Adrenaline Face Mask. For all exercise testing, the included disposable filter was secured to the inside of the ESFM with safety pins.

A within-subjects, crossover, repeated measures design where participants were randomly assigned to ESFM and unmasked conditions was used. Pre-trial instructions included refraining from any form of exercise, caffeine, or alcohol on the day of the trial. Participants were instructed not to use short-acting β2-agonists within 24 h prior to a test and not to use corticosteroids and long-acting β2-agonists 72 h prior to a test. The ESFM used was manufactured by Outdoor Research (Adrenaline Sports Face Cover Kit, Outdoor Research, Seattle, WA, USA) and was specifically marketed for use during “high-exertion workouts and labor-intensive activities” [10]. The mask is constructed of 100% polyester and includes both a wire nose bridge and adjustable ear loops [10]. As per manufacturer instructions, the ESFM was used with the included disposable filter, which was secured with safety pins to prevent the filter from shifting during the exercise trial (Figure 1). This face mask is classified as an ASTM Level II barrier face covering and thus elicits an airflow resistance of 0.5–1.5 cmH_2_O when measured at a continuous flow rate of 85L/min [22].

The exercise protocol was a discontinuous graded exercise test (GXT) performed on a treadmill (FMTK72509 Incline Trainer, Freemotion Fitness, Logan, UT, USA). The test consisted of up to nine 5 min stages, each performed at a constant velocity. The prolonged nature of this GXT protocol was designed to maximize osmotic stress on the airways in addition to amplifying any perceptual sensations experienced. The treadmill velocity for the first stage was set to replicate a running pace of 7:00 min per kilometer (min/km) with pace increases of 0:30 min/km until volitional exhaustion was reached, while the gradient was held constant at 1%. Subjective and respiratory function measures were taken between each consecutive stage, necessitating a 1 min standing period between stages. All exercise testing was conducted in an indoor, climate-controlled exercise laboratory, where the temperature and humidity were held constant with an ambient temperature of 19–20 °C and an estimated absolute humidity of 2–8 mg H_2_O/L of air. These environmental conditions are considered typical for indoor exercise [23] and have previously been identified as adequate for the provocation of EIB, with an absolute humidity of less than 10 mg H_2_O/L [24].

### 2.3. Measures

Both heart rate and breathing frequency were continuously monitored during exercise using an Equivital monitoring vest (EqO_2_+ LifeMonitor, Equivital, New York, NY, USA) streamed wirelessly into electronic charting software (LabChart 8, ADInstruments, Colorado Springs, CO, USA). Arterial oxygen saturation (SpO_2_) was assessed continuously via a Nellcor pulse oximeter (Nellcor N-600x, Medtronic, Minneapolis, MN, USA) utilizing a forehead probe affixed above the left eyebrow. Time to exhaustion was also assessed in both exercise conditions, with inter-stage rest periods being omitted.

Perceptual measures were acquired at rest, following each stage of the GXT, and at exercise termination. The Dalhousie Dyspnea Scales [25] assessed respiratory sensations, which is a 4-item tool, each rated on a 7-point pictorial scale. These sensations included (a) breathing discomfort, (b) chest tightness, (c) throat tightness, and (d) leg discomfort. Air hunger and perceived WOB were also assessed [26]. These 7-point scales consist of verbal cues ranging from “None” (1) to “Extreme” (7). Finally, the Borg RPE scale [27] assessed global perception of exertion on a 15-point scale. Following each running trial, participants were asked to rate their session RPE on the Borg CR-10 scale [27].

Respiratory measures consisted of forced vital capacity maneuvers performed into a calibrated portable spirometer (Spirodoc, MIR, Roma, Italy), which were conducted at baseline, following each stage, following exercise termination, and serially post-exercise. The participants were instructed to complete a forced vital capacity maneuver based on American Thoracic Society guidelines for spirometry [28]. Several standard respiratory function measures were automatically outputted following the completion of each maneuver, including forced expiratory volume in 1 s (FEV_1_), forced vital capacity (FVC), FEV_1_/FVC ratio, peak expiratory flow (PEF), forced expiratory flow between 25% and 75% of FVC (FEF_25–75_), and forced expiratory flow at 50% of FVC (FEF_50_).

During the exercise protocol, single FVC maneuvers were performed in a standing position following the completion of each stage of the GXT. To determine the EIB status of participants, spirometry was also performed serially following the graded exercise assessment at 3, 6, 10, 15, 20, and 30 min post-exercise. The highest FEV_1_ from each time point was included in the analysis, with a ≥10% post-exercise reduction from baseline at any post-exercise test indicating EIB [29].

### 2.4. Data Preparation

All analyses for this research project were conducted in SPSS Statistics (SPSS Statistics 28, IBM, Armonk, NY, USA) using an alpha value of 0.05 as a threshold for indicating significant differences.

To account for differences in exercise performance, submaximal physiological and perceptual responses were normalized across several discrete exercise intensities. The final stage completed under both experimental conditions for each subject was designated their “peak equivalent velocity”. Subsequently, stages approximating 70, 80, and 90% of this velocity were identified for each participant and were included in the analyses of submaximal responses. Physiological measures averaged over the final 30 s of each stage were used to indicate the physiological responses to each intensity. Perceptual measures collected immediately following the completion of each stage indicated submaximal perceptual responses. Due to technical issues with data collection equipment, submaximal SpO_2_ data for two participants were excluded from the analysis (n = 22). Additionally, breathing frequency data could not be obtained for two participants due to fit of the monitoring vest (n = 22).

### 2.5. Statistical Analysis

A linear mixed-effect model was applied to subjective and physiological measures assessed at numerous submaximal exercise intensities during the GXT (condition X submaximal intensity). The model consisted of random intercepts with fixed slopes. The fixed effects examined included condition (ESFM vs. unmasked) and exercise intensity (70, 80, 90, and 100% peak equivalent velocity), with participants being treated as a random effect. The main effects of each of the fixed effects were assessed in addition to their interaction. For significant interactions, paired-samples *t*-tests for each exercise intensity were used to compare masked and unmasked exercise conditions.

Scales administered immediately following participant termination under each condition indicated maximal responses. For physiological measures, the 30 s average preceding exercise termination indicated maximal responses. Maximal responses were assessed via a paired-sample *t*-test (between conditions). Normality was assessed using a Shapiro–Wilk test where normality violations or outliers indicated the use of either a Wilcoxon Rank Sign test or a Sign test [Laerd Statistics, 2023]. Due to technical issues, SpO_2_ data were not captured for five participants at termination under both conditions (n = 19).

All values derived from spirometry were analyzed as a delta percentage of the pre-exercise baseline. These included FEV_1_, FVC, FEV_1_/FVC, PEF, FEF_25–75_, and FEF_50_. For submaximal respiratory responses, all stages from the start of the GXT protocol until the point of substantial data attrition (>10%) due to participant dropout were analyzed via a two-way repeated measures analysis of variance. To examine respiratory responses to intense exercise, pairwise comparisons of spirometry values were made with both measures collected at termination and with measures collected at 100% peak equivalent velocity. If a technically adequate spirometry maneuver was not achieved for a given submaximal exercise intensity, the values from the previous FVC maneuver were carried forward.

## 3. Results

### 3.1. Descriptives

Descriptive variables are shown in Table 1. Baseline respiratory function was not significantly different between the two experimental conditions for any of the spirometry measures analyzed (Table 1). One participant was excluded from all spirometry analyses because they were unable to perform technically proficient FVC maneuvers in one of their experimental trials (n = 23). Of the remaining 23 participants with valid respiratory function measures, participants differed in their EIB status. A total of three participants were EIB+ under both experimental conditions (one female), two were EIB+ in the masked condition only (one female), and one was EIB+ in the unmasked condition only (male). Seventeen participants (nine female) did not have a 10% or greater reduction in FEV_1_ post-exercise under either condition (EIB-).

### 3.2. Submaximal Physiological and Perceptual Responses

**Figure 2:**
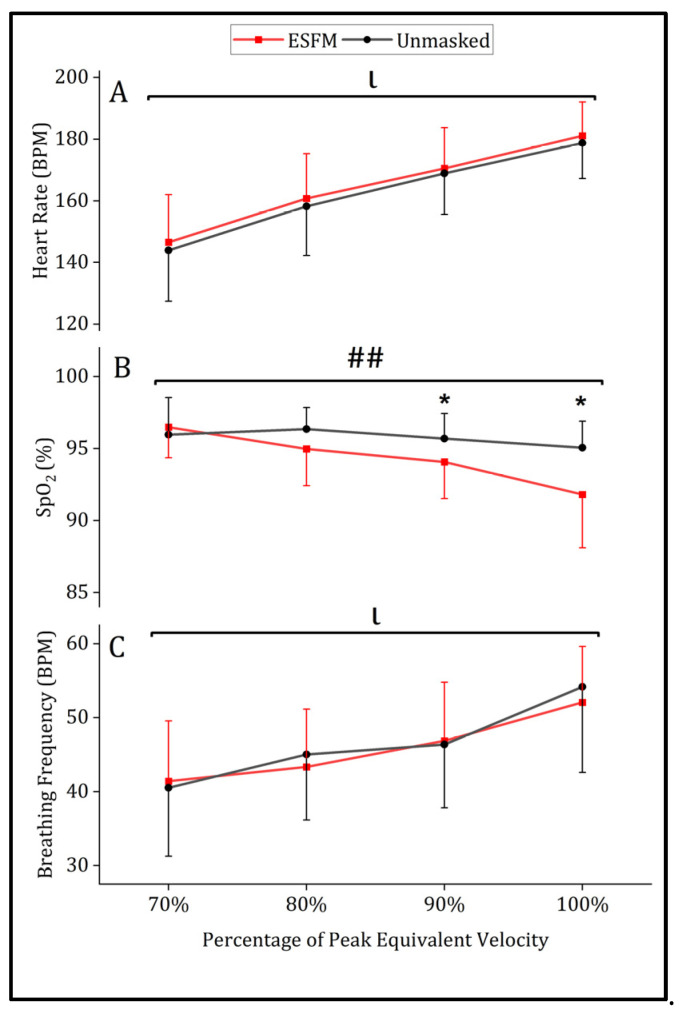
Mean (SD) physiological responses to treadmill running with (red) and without (gray) an ESFM for (**A**) heart rate in beats/min, (**B**) arterial oxygen saturation (%) and (**C**) breathing frequency in breaths/min. ## significant interaction effect (intensity x condition). ι significant main effect (intensity). * significant pairwise comparisons (condition) SpO_2_, arterial oxygen saturation (%).

A significant main effect of intensity was observed for all physiological measures analyzed (*p* < 0.001). Use of an ESFM did not significantly alter submaximal heart rate, with no significant main effect of condition (ESFM–Unmasked = +2.4 BPM, F = 0.735, *p* = 0.394) or interaction between intensity and condition (F = 0.025, *p* = 0.875) observed (Figure 2A). No differences in submaximal breathing frequency were observed for the main effect of the condition (ESFM–Unmasked = −0.5 breaths/min, F = 0.571, *p* = 0.452) or interaction (F = 1.298, *p* = 0.259; Figure 2C). SpO_2_ differed between conditions overall (ESFM–Unmasked = −1.2%, F = 3.995, *p* = 0.049), with an interaction (F = 16.141, *p* < 0.001) indicating that SpO_2_ was significantly decreased in the ESFM condition at 90, 100%, and termination (Figure 2B).

**Figure 3:**
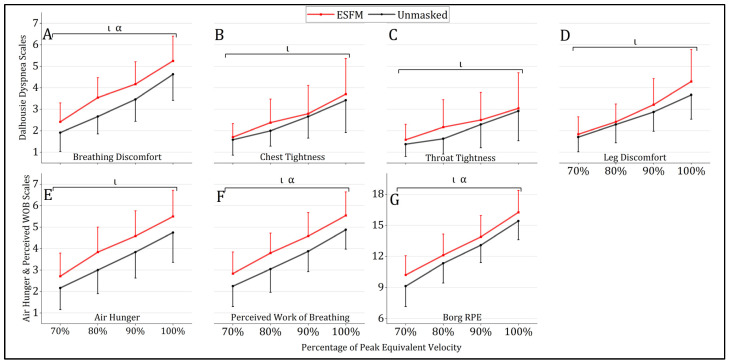
Mean (SD) perceptual responses to treadmill running with (red) and without (gray) an ESFM. (**A**) breathing discomfort “how hard does your breathing feel”, (**B**) chest tightness “how tight does your chest feel”, (**C**) throat tightness “how tight does your throat feel”, (**D**) leg discomfort “how do your legs feel”, (**E**) air hunger “discomfort caused by urge to breathe”, (**F**) perceived work of breathing, “sensations of work to satisfy breathing requirements” and (**G**) rating of whole body perceived exertion. α significant (*p* < 0.05) main effect (condition); ι significant (*p* < 0.05) main effect (intensity).

Use of an ESFM elevated several perceptual measures during submaximal exercise, with a significant main effect of condition being observed for breathing discomfort (ESFM–Unmasked = +0.6, F = 4.978, *p* = 0.029), perceived WOB (ESFM–Unmasked = +0.7, F = 7.501, *p* = 0.008), and RPE (ESFM–Unmasked = +0.9, F = 4.780, *p* = 0.032) (Figure 3). An ESFM did not alter the degree of leg discomfort, chest tightness, throat tightness, or air hunger during submaximal intensity exercise compared to the unmasked condition, with no main effect of condition observed (*p* > 0.05). A significant main effect of intensity was observed for all perceptual measures analyzed (*p* < 0.001). No significant interaction effects were observed for any perceptual measures analyzed.

### 3.3. Maximal Physiological and Perceptual Responses

A significantly lower SpO_2_ was observed at termination in the ESFM condition (89.7%) when compared to the unmasked condition (93.4%, t (18) = 3.413, *p* = 0.003). Maximal heart rate at exercise termination was not different between ESFM (184.9 BPM) and unmasked conditions (185.7 BPM, t (23) = 0.669, *p* = 0.510). The mean breathing frequency at exercise termination was not different between ESFM (61.1 breaths/min) and unmasked conditions (63.1 breaths/min, t (21) = 0.991, *p* = 0.333).

The use of an ESFM was found to significantly elevate both air hunger and perceived WOB at termination. A total of 11 of 24 participants reported higher perceived air hunger at termination while wearing an ESFM, with only a single participant reporting reduced air hunger in the ESFM condition (sign test, *p* = 0.006). Perceived WOB at termination was higher in the ESFM condition for 12/24 participants, equal in 11 participants, and lower in 1 participant (sign test, *p* = 0.003). No significant differences in perceptual responses to maximal exercise with an ESFM were observed for RPE, session RPE, breathing discomfort, leg discomfort, chest tightness, or throat tightness (Table 2).

### 3.4. Respiratory Responses

**Figure 4:**
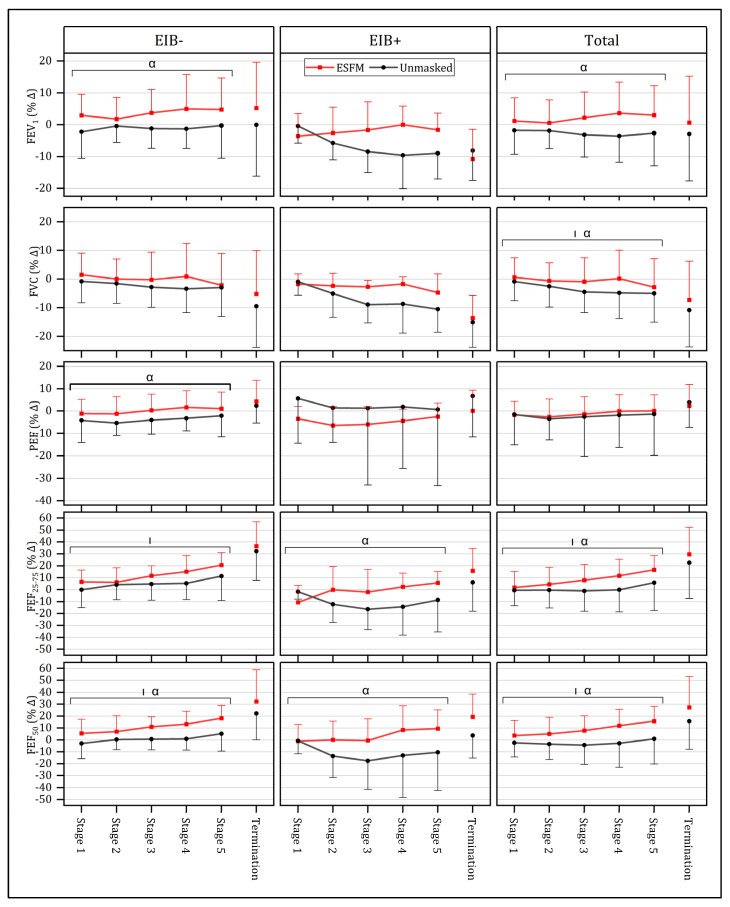
Mean (SD) respiratory responses to submaximal (Stages 1–5) and maximal (Termination) intensity exercise both with (red) and without an ESFM (gray) as a percentage change from pre-exercise baseline, with participants with (EIB+) and without (EIB-) exercise-induced bronchoconstriction being compared. α significant (*p* < 0.05) main effect (condition); | significant (*p* < 0.05) main effect (intensity); FEV_1_, forced expiratory volume in 1 s; FVC, forced vital capacity; FEF_25–75_, forced expiratory flow from 25% to 75% vital capacity; FEF_50_, forced expiratory flow at 50% vital capacity.

Submaximal respiratory responses were compared across conditions for the first five stages of the GXT, as this was the point to which near-complete data were available prior to attrition due to test termination (n = 22). No significant interaction effect between intensity and condition was identified for any of the spirometry measures assessed (Figure 4). Overall (n = 22), a significant main effect for condition was observed across several spirometry measures, including FEV_1_ (F = 19.20, *p* < 0.001), FVC (F = 4.49, *p* = 0.046), FEF_25–75_ (F = 9.05, *p* = 0.007), and FEF_50_ (F = 20.00, *p* < 0.001), with use of an ESFM improving respiratory function (Figure 4). PEF and FEV_1_/FVC were not significantly different between the ESFM and unmasked conditions_._ When individuals identified as EIB+ (n = 6) were analyzed separately, a significant main effect of condition was observed in both FEF_25–75_ (F = 8.46, *p* = 0.033) and FEF_50_ (F = 10.88, *p* = 0.022). In individuals who were EIB- (n = 16), a significant main effect of condition was observed in FEV_1_ (F = 12.634, *p* = 0.003), PEF (F = 5.25, *p* = 0.037), and FEF_50_ (F = 10.74, *p* = 0.005) (Figure 4).

Given the presence of outliers, non-parametric pairwise comparisons were performed between conditions for the respiratory function comparisons (n = 23). When respiratory function at exercise termination was assessed, no significant differences between the ESFM and unmasked condition were observed in FEV_1_, FVC, FEV_1_/FVC, PEF, FEF_25–75_, or FEF_50_ at termination, expressed as a percentage of the pre-exercise baseline. The last stage completed in both conditions was also compared to provide a fair estimate of within-exercise respiratory function with matched exercise duration. In the ESFM condition, mid-expiratory flow measures of respiratory function were increased. Specifically, FEF_25–75_ (*p* = 0.010) and FEF_50_ (*p* = 0.012) were increased (17/23 participants had increased FEF_25–75_ and 16/23 participants exhibited greater FEF_50_) when the ESFM was worn.

### 3.5. Exercise Performance

A total of 21 out of 24 participants had a longer time to exhaustion in the unmasked condition, and 3 performed better in the ESFM condition. The median decrease in test performance as assessed through time to exhaustion was statistically significant (−150.5 s), with a shorter median time to exhaustion observed when an ESFM was worn (2163.0 s) versus when no mask was worn (2216.5 s), sign test, *p* < 0.001.

## 4. Discussion

The use of a face mask to protect against airborne viruses and pollutants such as dust is common in a variety of daily activities where exposure may be certain. However, little research has examined how a face covering alters within- and post-exercise respiratory function and perceptual responses to aerobic exercise, especially in a normal indoor ambient setting. Thus, our aim was to explore the benefits and drawbacks of wearing an ESFM during aerobic exercise and whether this ESFM might alter post-exercise respiratory function. Our results found that an ESFM is protective of the lung, improving within-exercise respiratory function. However, these benefits need to be balanced with the degree of perceptual and physiological burden, elevating multiple sensations of dyspnea and inducing significant reductions in SpO_2_ at both submaximal and maximal exercise intensities. This research provides important information for high-ventilation athletes because it documents the impaired performance resulting from altered perceptual and physiological responses, which may undercut the respiratory-protective benefits. The meaning of these results is discussed in more detail below.

### 4.1. Physiological Impact

Our results reflect an altered physiological response in the masked condition, with an insignificant but consistently increased heart rate across all submaximal exercise intensities (*p* > 0.05) in the absence of significant changes in breathing frequency. A recent meta-analysis [2] also showed that a PFM did not significantly modify breathing frequency during either steady-state (−0.3 breaths/min) or maximal intensity exercise (−1.4 breaths/min). The mean heart rate elevation observed across all submaximal intensities with an ESFM in our study (+2.4 BPM, *p* > 0.05) was also similar to that reported in a meta-analysis by Zheng et al. [2] for steady-state exercise with a PFM (+2.7 BPM). Given that the estimated airflow resistance for the mask used in this study is 0.5–1.5 cmH_2_O, we would suggest that the resistance imposed by the ESFM was not substantial enough to depress breathing frequency or modify breathing patterns. This aligns with previous research, which found that high-resistance metabolic systems (~2.5 cmH_2_O at 90 L/min) significantly alter breathing frequency when compared to a low-resistance alternative (~0.8 cmH_2_O at 90 L/min) [31]. In addition, Marek et al. [32] found that a similar type of mask also had negligible differences in breathing frequency and tidal volume during heavy to very heavy work, although their results need to be interpreted in the context of their physical breathing apparatus, as discussed below. We also cannot discount that alterations in tidal volume or respiratory cycle time maintained breathing frequency despite the increased resistance that may have been imposed in the masked condition. To understand if fitness had an influence on breathing frequency between conditions, post hoc analysis of the best-performing participants (i.e., greatest running velocities, n = 4) was examined. In this cohort, breathing frequency at termination was not significantly different between conditions (ESFM = 62.0 breaths/min, unmasked = 61.1 breaths/min) either. These participants had the highest exercise intensity and likely high absolute ventilatory demands. Based on our data, we can at least surmise that breathing frequency associated with significant ventilatory demands is not significantly altered by an ESFM. It remains to be determined if other aspects of breathing dynamics are altered at heavy workloads with high ventilatory demands when an ESFM is worn, and should be an avenue for future research directions.

Despite this, SpO_2_ was reduced in the ESFM condition when compared to the unmasked condition, although the reasons for this reduced SpO_2_ are unclear. In unmasked conditions, arterial desaturation is often observed in aerobically fit individuals at near-maximal and maximal exercise intensities [33]. First, provided the mean SpO_2_ at exercise termination was 3.7% lower in the ESFM condition when compared to unmasked, it appears an ESFM exacerbates exercise-induced SpO_2_ reductions. As the use of a PFM increases both dead space and breathing resistance [6], differences in SpO_2_ between conditions may have stemmed from several different underlying sources. Elevated dead space ventilation can modify inspired gas tensions, in turn elevating inspired CO_2_ and depressing inspired O_2_, although the extent of this change in the context of small dead space volumes of ~100 mL is debatable [34]. A similar type of “community mask” has shown that the resistance associated with this type of mask had no difference on SpO_2_ despite elevated PaCO_2_ and decreased PaO_2_ during heavy to very heavy work [32]. However, a key difference between studies exists where, in our study, a full mask was worn as per the manufacturer’s recommendations compared to a one-way valve mouthpiece worn off the face in the other study [32]. Thus, these results elucidate that a full mask does reduce SpO_2_, although why this may be is beyond the scope of this project. We would hypothesize that reduced SpO_2_ is due to inadequate hyperpnea because of either blunted chemosensitivity or a mechanical ventilatory limitation. If inadequate hyperpnea did result in reductions of PaO_2_ when combined with an accentuated rightward shift of the oxyhemoglobin disassociation curve, it may have been large enough to induce reductions in SpO_2_. This conjecture is supported by the existing literature, which indicates that PFM-induced hypoventilation elevates end-tidal carbon dioxide when a PFM is worn during both maximal and submaximal exercise [2].

The 3.7% mean reduction in SpO_2_ at exercise termination likely has pragmatic implications for some aerobically fit individuals. The implication is that the absolute SpO_2_ in the ESFM condition was <90%, which, for some individuals, may influence their ability to continue to exercise or achieve a higher VO_2_ max in a graded exercise test [35]. Given that treadmill running, when compared to cycle ergometry, induces greater SpO_2_ changes [35], our methods provide a sufficient exercise stimulus to clearly understand how an ESFM might alter SpO_2_. How the ESFM may alter the acid–base balance or arterial O_2_ and CO_2_ tension as contributing factors to reduced SpO_2_ is not known. Future investigations might seek to understand other mechanisms that cause reduced SpO_2_ in the ESFM condition.

### 4.2. Perceptual Impact

Previous research has often limited perceptual measures to RPE and dyspnea [2] or has used a combination of a metabolic collection mask with a PFM [3,4,5], which is not indicative of real-world exercise conditions. Other studies have found increased dyspnea both with [34] and in the absence of [36] significant physiological differences, which highlights that perceptual differences are not necessarily tied to clear physiological changes. Thus, our research focused on understanding dyspnea in a number of discrete domains, and the significant findings are discussed in more detail below.

Under the umbrella of different dyspnea measures, air hunger and perceived WOB were the only two perceptual measures that were significantly elevated at termination when an ESFM was worn, and these max responses were likely due to the elevated submaximal increases (air hunger = +0.7, perceived WOB = +0.7) that persisted until termination (air hunger = +0.5, perceived WOB = +0.5) in the ESFM condition (Figure 2). These two measures are distinct contributors to dyspnea yet likely differ in their physiological sources [26]. Sensations of air hunger are closely tied to the “balance between respiratory drive arising from chemoreceptors and other inputs versus respiratory tidal excursions reported by mechanoreceptors” [37]. Provided that elevated end-tidal carbon dioxide has been consistently reported during exercise when a PFM is worn [2], PFM-induced changes in blood gas tensions may be partially responsible for elevations in air hunger observed during submaximal and maximal intensity exercise. Additionally, hypoxemia may have also contributed to elevations in this sensation, particularly at higher exercise intensities over 90% peak equivalent velocity, where SpO_2_ was significantly decreased in the ESFM condition (Figure 2). On the other hand, although perceived WOB during exercise is closely associated with increases in minute ventilation under typical exercise circumstances [26], resistance imposed by the ESFM may further elevate this domain of dyspnea during exercise through the elevation of WOB for a given exercise intensity. In the context of ESFM use, elevated resistance was likely the primary contributor to the observed differences in perceived WOB, given its direct influence on elevating WOB [38]. Yet, the increased perceived WOB/air hunger is seemingly disproportionate to the relatively small potential breathing resistance and dead space imposed by a PFM [6], and thus, other factors likely contributed to the observed elevations in these sensations. It is possible that the temperature/humidity of the microenvironment under the mask was also a contributing factor. In fact, a cloth-based fabric mask elevates temperature by about 3.7 °C and relative humidity as much as 40% during heavy to very heavy work and the authors ascribed that a cloth-based mask holds significant moisture, which may have altered comfort [32]. In our study, the mask was 100% synthetic but of a similar shape; thus, we would assume that elevated temperature and humidity occurred compared to unmasked however the magnitude of the increase would be less than others [32]. We would also offer that insufficient blinding of participants may have also contributed to the increased perceptual burden associated with the ESFM condition. This is clearly an impossible design to blind someone to wearing a mask and we accept that just knowing you are wearing a mask could have altered the reported perceptual burden in some participants. We think this is an important area of continued research, because how the relative combination of resistance, dead space, and other factors interact to alter perceptual burden remains to be fully elucidated.

In addition to perceived WOB and air hunger, the Dalhousie Dyspnea Scales and Borg RPE Scale provided further insight into perceptual sensations associated with the ESFM. Both breathing discomfort and RPE were significantly elevated across submaximal intensities but not at exercise termination. Provided that test performance was significantly impaired when an ESFM was worn, with 54% of participants terminating at a slower velocity in the ESFM condition, it could be hypothesized that termination occurred sooner but at an equivalent level of global perceptual discomfort when an ESFM was worn. Given that chest tightness and throat tightness are specific qualities of dyspnea often associated with bronchoconstriction and reductions in airway caliber [39], it stands to reason that the ESFM condition might reduce chest or throat tightness because respiratory function was improved during exercise (as indicated by spirometry). Yet, as ascertained above, perceived WOB was elevated, which might induce more chest and throat tightness independent of changes in respiratory function. Overall, the combination of perceptual findings reveals the complicated relationship between different domains of dyspnea and highlights why future research should aim to disentangle these perceptual and physiological factors in the context of PFM use.

### 4.3. Respiratory Impact

Significant differences in spirometry measures were observed during submaximal exercise when the ESFM and unmasked conditions were compared. The use of FVC maneuvers is one of the most common methods used to assess changes in airway caliber associated with exercise and is a valid reflection of airway obstruction [40]. The respiratory measures taken during the exercise protocol suggest that the use of an ESFM may help to maintain baseline respiratory function in hyperresponsive individuals and promote bronchodilation in individuals without EIB. For example, overall, both FEF_25–75_ and FEF_50_ were significantly elevated at 100% peak equivalent velocity when an ESFM was worn. Given that decreased mid-expiratory flow measures are commonly used as indices of small airway obstruction [41], improved FEF_50_ and FEF_25–75_ during the ESFM condition suggests that the extent to which small airways were brought into the conditioning process was likely less than in the unmasked condition. Thus, when ventilation is increased, the use of a face mask may act to promote increases in small airway caliber, likely due to reduced osmotic stress [42], which protects those with no known EIB and those who might have EIB [43]. Given the virulent effect dry air has on eliciting osmotic stress in the airway, research has shown that a cold-weather heat and moisture exchanger can be effective in attenuating reductions in respiratory function during exercise [12,16]. However, our findings illustrate that a high-performance fabric mask worn in warm, dry conditions can also improve respiratory function.

Although the benefits of wearing a face covering during exercise have been largely promoted to those with EIB [44], there may also be acute benefits for those without EIB. In this cohort (n = 16), FEV_1_, PEF, and FEF_50_ were significantly elevated during submaximal intensity exercise when a mask was worn, suggesting the use of an ESFM promotes additional bronchodilation when worn during exercise. There is evidence to suggest that increasing the absolute water content of inspired air can also promote bronchodilation in healthy individuals without asthma [45]. Although the specific mechanism for this occurrence was not hypothesized, the ESFM may act by delaying or preventing the small airways from being used to condition relatively dry inspired air. Research examining the responses of healthy athletes exercising in cold, dry environmental conditions has also demonstrated the ability of a heat and moisture exchange device to improve post-exercise respiratory function [13]. This is also suggested by research on competitive winter sports athletes, where significantly higher levels of inflammatory exudate in the airway were observed, with authors recommending limiting osmotic and thermal stress often experienced as part of training [46]. By limiting the extent to which peripheral airways are required to condition inspired air, the use of an ESFM could have a respiratory-protective benefit, acutely promoting increased airway caliber and potentially limiting airway hyperresponsiveness. This likely results in improved long-term lung health, where long-term remodeling might occur not just in cold-weather athletes [46] but also in individuals who habitually exercise in dry climates. Given the consistent findings of improved respiratory function across a range of exercise intensities found in this study, we would recommend that an ESFM be used for aerobic exercise in indoor ambient conditions. The only caveat to this would be that if the perceptual burden with an ESFM is altering exercise motivation, the ESFM will be removed or the intensity of exercise will be reduced.

### 4.4. Performance Impact

Provided that 88% of participants performed worse in the ESFM condition, as shown by time to exhaustion, it is worth considering why this may be the case. Considering that RPE and breathing discomfort were not significantly different between experimental conditions at termination, it could be speculated that voluntary exercise termination occurred at the point at which discomfort exceeded individual motivation to continue [47]. As the ESFM elevated RPE (mean elevation = +0.9) and breathing discomfort (mean elevation = +0.6) across submaximal exercise intensities, the threshold at which exercise was no longer perceptually tolerable likely occurred sooner in the GXT under the ESFM condition.

Although collective research findings suggest a lack of performance impairment when cloth or surgical masks are worn [2], the utilization of a longer GXT protocol consisting of 5 min stages likely accentuated the performance impairment in this study. Additionally, given that our sample consisted of aerobically fit individuals, including many endurance athletes, even small reductions in the maximal flow volume envelope induced through mask resistance have the potential to expedite a ventilatory limitation, provided that endurance athletes are more likely to be flow limited at peak exercise when compared to healthy individuals [48]. Regardless of the mechanism underpinning impaired performance, this finding suggests that willingness to wear an ESFM, particularly in athletic competitions, is likely low. Specifically, the results of our study suggest that performance in prolonged, high-intensity aerobic exercise events would be impaired in aerobically fit individuals when an ESFM is worn.

### 4.5. Limitations

It could be argued that the exercise provocation of this study may have been insufficient to induce bronchoconstriction in all participants with EIB, leading some to be misclassified as EIB-. As guidelines for EIB exercise provocation recommend that high ventilation be rapidly achieved and maintained for 6–8 min, it is possible that the exercise intensity in the first stages of the GXT may have increased too slowly for immediate recruitment of smaller airways to take place [24]. Given that the first stages likely elicited relatively low minute ventilation, particularly in more aerobically fit participants, it is possible that some participants were misclassified as EIB-_._ Despite this, the prolonged nature of this protocol may have been necessary to elicit within-exercise changes in respiratory function, which have previously been reported in EIB+ cross-country skiers [17]. We also have a mixed-sex cohort that may have altered the results based on sex-specific responses to either condition. We would have liked to make a sex-based comparison; however, we felt we were underpowered to make this a legitimate comparison. Future research should look at sex-based differences between ESFM and no mask to elucidate potential differences.

## 5. Conclusions

Specifically, based on these results, the use of an ESFM during exercise is appropriate in healthy and aerobically fit individuals. We found elevated perceptual burden with some physiological changes, although the specific link between perceptual and physiological changes is not entirely clear in our study. Despite the heightened perceptual discomfort when wearing an ESFM during exercise, improved within-exercise respiratory function indicates that ESFMs can benefit respiratory function. Despite this benefit, the reduction in time to exhaustion observed would suggest limited utility in actual endurance sports competition, especially if any heightened sensations of air hunger or perceived WOB were apparent. Thus, we suggest that lower-intensity aerobic exercise might be the best usage of an ESFM in most individuals. Specifically, a person might determine the specific intensity threshold where the increased ventilation might cause a significant perceptual burden and then wear an ESFM at exercise intensities below this threshold. Given that ESFMs act as a “double-edged sword” when worn during exercise, imposing a perceptual burden and potentially reducing performance while promoting respiratory function, future research should focus on the development and evaluation of face coverings that impose minimal perceptual and physiological burden during exercise while still providing respiratory-protective benefits to the user.

## Figures and Tables

**Table 1:** Participant characteristics.

		Sex		EIB Status		
		Female (n = 11)	Male (n = 13) *	EIB+ (n = 6)	EIB- (n = 17)	Total (n = 24) **
		Mean (SD)	Mean (SD)	Mean (SD)	Mean (SD)	Mean (SD)
	Age (Years)	26 (7)	29 (8)	27 (7)	28 (8)	28 (8)
	Height (cm)	167 (7)	188 (7)	186 (12)	175 (12)	178 (13)
	Weight (kg)	62.4 (6.0)	81.5 (8.2)	73.6 (10.9)	69.9 (11.4)	71.9 (12.1)
	BMI (AU)	22.5 (2.8)	23.0 (1.9)	21.3 (0.9)	23.0 (2.4)	22.8 (2.4)
	Years of Competitive Endurance Sport	9 (6)	15 (7)	15 (5)	10 (8)	12 (7)
Unmasked	FeNO (PPB)	14 (5)	63 (54)	74 (72)	27 (25)	40 (46)
	Post-exercise FEV_1_ Nadir (% Δ)	−1.2 (7.3)	−5.8 (7.7)	−11.6 (9.8)	−0.8 (4.4)	−3.6 (7.7)
	FEV_1_ (L) *% pred*	3.42 (0.58) 99 (13)	5.11 (0.94) 100 (14)	4.78 (1.43) 97 (13)	4.14 (1.05) 101 (14)	4.30 (1.16) 100 (13)
	FVC (L) *% pred*	4.32 (0.66) 108 (14)	6.99 (1.68) 112 (25)	6.38 (1.94) 107 (19)	5.48 (1.84) 111 (21)	5.71 (1.86) 110 (20)
	PEF (L/s)	7.16 (0.74)	9.92 (1.6)	9.23 (2.23)	8.38 (1.75)	8.60 (1.87)
	FEF_25–75_ (L/s) *% pred*	3.41 (0.86) 87 (18)	4.35 (1.18) 86 (18)	4.70 (1.41) 94 (19)	3.62 (0.89) 83 (17)	3.90 (1.12) 86 (18)
	FEF_50_ (L/s)	4.22 (1.20)	4.95 (1.19)	5.35 (1.37)	4.34 (1.09)	4.60 (1.23)
	FEV_1_/FVC (%) *% pred*	79.6 (9.8) 92 (10)	74.3 (7.7) 90 (9)	76.3 (12.9) 91 (14)	77.0 (7.7) 91 (8)	76.8 (9.0) 91 (10)
ESFM	FeNO (PPB)	15 (7)	52 (41)	58 (55)	26 (20)	34 (35)
	Post-exercise FEV_1_ Nadir (% Δ)	−0.6 (9.4)	−4.6 (9.4)	−14.5 (5.9)	+1.5 (6.3)	−2.7 (9.4)
	FEV_1_ (L) *% pred*	3.46 (0.60) 100 (13)	5.06 (1.08) 99 (16)	5.11 (1.62) 104 (19)	4.00 (0.88) 98 (13)	4.29 (1.19) 100 (14)
	FVC (L) *% pred*	4.32 (0.70) 108 (16)	6.86 (1.49) 110 (20)	6.47 (2.23) 108 (23)	5.35 (1.50) 109 (16)	5.64 (1.73) 109 (18)
	PEF (L/s)	7.23 (0.98)	9.92 (1.63)	9.90 (2.34)	8.19 (1.58)	8.63 (1.91)
	FEF_25–75_ (L/s) *% pred*	3.27 (0.90) 84 (20)	4.32 (1.57) 85 (25)	5.04 (1.97) 100 (28)	3.39 (0.80) 79 (18)	3.82 (1.38) 84 (22)
	FEF_50_ (L/s)	3.80 (1.17)	4.86 (1.54)	5.36 (1.99)	4.00 (1.06)	4.36 (1.45)
	FEV_1_/FVC (%) *% pred*	80.0 (7.5) 93 (8)	74.5 (8.0) 90 (9)	80.4 (9.3) 96 (9)	76.0 (7.6) 90 (8)	77.1 (8.1) 92 (9)
Cohen’s d Effect Sizes for Unmasked vs. ESFM		d	d	d	d	d
	FeNO (PPB)	0.164	0.229	0.2497	0.044	0.146
	Post-exercise FEV_1_ Nadir (% Δ)	0.071	0.139	0.358	0.423	0.104
	FEV_1_ (L)	0.067	0.049	0.215	0.144	0.008
	FVC (L)	0.029	0.081	0.043	0.077	0.038
	PEF (L/s)	0.080	0.000	0.293	0.113	0.015
	FEF_25–75_ (L/s)	0.159	0.022	0.198	0.271	0.063
	FEF_50_ (L/s)	0.354	0.065	0.005	0.316	0.178
	FEV_1_/FVC (%*)*	0.045	0.025	0.364	0.131	0.035

* n = 12 for spirometry values. ** n = 23 for spirometry values; data reported as mean (SD) SD, standard deviation. BMI, body mass index; FENO, fractional expired nitric oxide; FEV_1_, forced expiratory volume in 1 s; FVC, forced vital capacity; FEF_25–75_, forced expiratory flow from 25% to 75% vital capacity; FEF_50_, forced expiratory flow at 50% vital capacity; FEV_1_/FVC, ratio of FEV_1_ compared to FVC; *% pred*, predicted values derived from 2012 Global Lung Function Equations [30]. No significant differences in baseline spirometry values between ESFM and unmasked conditions (*p* > 0.05). Cohen’s d provided to show effect size for the standardized mean difference between masked and unmasked conditions.

**Table 2:** Perceptual and physiological responses to maximal intensity exercise with and without an ESFM.

		ESFM		Unmasked		Mean Difference	
	n	Mean (SD)	Median	Mean (SD)	Median	(ESFM-CON)	*Test* *p*
Air Hunger (1–7)	24	6.5 (0.8)	7.0	6.0 (1.0)	6.0	+0.5	Sign 0.006 *
Perceived Work of Breathing (1–7)	24	6.5 (0.7)	7.0	6.0 (0.93)	6.0	+0.5	Sign 0.003 *
Breathing Discomfort (1–7)	24	6.2 (1.0)	6.0	6.1 (1.0)	6.0	+0.1	Wilcoxon 0.527
Chest Tightness (1–7)	24	4.3 (1.6)	4.5	4.4 (1.7)	4.5	−0.1	Wilcoxon 0.572
Throat Tightness (1–7)	24	3.8 (2.2)	3.0	4.0 (1.7)	4.0	−0.1	Wilcoxon 0.730
Leg Discomfort (1–7)	24	5.0 (1.5)	5.5	5.1 (1.6)	5.0	0.0	Sign 1.000
Borg RPE (6–20)	24	18.8 (1.2)	19.0	18.4 (1.7)	19.0	+0.3	Wilcoxon 0.393
Session RPE (0–10)	24	6.5 (2.0)	7.0	6.6 (1.7)	7.0	−0.1	Wilcoxon 0.799
Heart Rate (BPM)	24	184.9 (10.0)	185	185.7 (12.2)	183	−0.8	*t*-Test 0.510
Breathing Frequency (Breaths/min)	22	61.1 (9.5)	61	63.1 (9.8)	63	−1.9	*t*-Test 0.333
SpO_2_ (%)	19	89.7 (5.1)	91	93.4 (3.9)	94	−3.7	*t*-Test 0.003 *

ESFM, exercise specific face mask; RPE, rating of perceived exertion; SpO_2_, arterial oxygen saturation. * significant difference between ESFM and unmasked conditions.

## Data Availability

Study data are available upon reasonable request.
